# Pasteurized *Akkermansia muciniphila* Muc^T^ reduces high-caloric diet-induced weight gain and alters bile acid and inflammatory profiles in dogs

**DOI:** 10.1128/spectrum.03226-25

**Published:** 2026-07-14

**Authors:** Harro M. Timmerman, Anneleen Segers, Jos Boekhorst, Dennis te Beest, Chi-Hsuan Sung, Amanda B. Blake, Jan S. Suchodolski, Willem M. de Vos

**Affiliations:** 1 Microbiome Innovation Consultancy, Rhenen, the Netherlands; 2 The Akkermansia Company, Mont-Saint-Guibert, Belgium; 3 Laboratory of Microbiology, Wageningen University 593528 , Wageningen, the Netherlands; 4 Laboratory of Host-Microbiome Interactions, Wageningen University 593528 , Wageningen, the Netherlands; 5 Biometris, Wageningen University & Research 4508, Wageningen, the Netherlands; 6 Gastrointestinal Laboratory, Texas A&M College of Veterinary Medicine & Biomedical Sciences 199062 , College Station, USA; 7 Human Microbiome Research Program, Faculty of Medicine, University of Helsinki 3835, Helsinki, Finland; University of Arkansas Fayetteville, Fayetteville, Arkansas, USA

**Keywords:** canine obesity, *Akkermansia muciniphila *Muc^T.^, high-caloric diet, bile acid metabolism, inflammation, multi-omics factor analysis

## Abstract

Obesity and excess body weight in companion animals represent significant and growing health concerns. Accumulating evidence indicates that obesity-associated metabolic and inflammatory dysfunction may be mitigated through dietary modulation of host-microbiome interactions. In this controlled dietary intervention study, healthy Beagle dogs were fed a commercially available high-caloric, protein-, and lipid-enriched diet at three times their daily energy requirement for 8 weeks and orally supplemented with either pasteurized *Akkermansia muciniphila* Muc^T^ or a placebo. Supplementation reduced weight gain and attenuated the high-caloric diet-induced expansion of *Peptacetobacter hiranonis* and *Collinsella* spp. compared to controls. Pasteurized *A. muciniphila*-treated animals exhibited reduced circulating pro-inflammatory cytokines and a marked decrease in fecal calprotectin levels, indicative of reduced mucosal inflammation. Longitudinal modeling identified treatment-associated alterations in inflammatory markers, while integrative multi-omics factor analysis (MOFA) and redundancy analysis (RDA) revealed coordinated changes across inflammatory, metabolic, and bile acid profiles that distinguished placebo- and pasteurized *A. muciniphila*-treated animals under high-caloric feeding conditions. These changes were accompanied by normalization of circulating bile acid profiles, particularly reductions in microbiome-derived secondary bile acids and oxo-derivatives associated with metabolic dysfunction. Together, these findings provide integrative insight into the immune-metabolic effects of pasteurized *A. muciniphila* Muc^T^ in dogs under high-caloric feeding conditions and support its potential as a dietary intervention to mitigate obesity-associated dysfunction in companion animals. As this is an exploratory proof-of-concept study conducted in a limited number of animals under controlled feeding conditions, further validation in larger and more diverse companion animal populations is warranted.

**IMPORTANCE:**

Obesity in pet dogs is increasingly recognized not only as a condition of excess body fat but as a chronic inflammatory disorder with profound metabolic consequences. This study demonstrates that dietary supplementation with pasteurized *Akkermansia muciniphila* Muc^T^ can counteract the adverse effects of a high-caloric diet in dogs by reducing body weight gain, preventing inflammation, and restoring bile acid homeostasis. These results highlight the therapeutic potential of gut-targeted nutritional strategies for managing obesity in companion animals and introduce *A. muciniphila* Muc^T^ as a safe, effective postbiotic with translational relevance for pet health.

## INTRODUCTION

Obesity is one of the most prevalent and pressing health concerns in companion animals, affecting an estimated 30–60% of pet dogs globally (1). Excessive adiposity in dogs often visibly impairs normal behavior and physical function. Obese dogs are frequently reported to exhibit reduced willingness to exercise, difficulty rising or moving comfortably, and a diminished capacity to engage in natural behaviors such as play, exploration, and social interaction. In addition to these welfare concerns, obesity is now recognized as a complex and chronic inflammatory condition that significantly impairs quality of life and predisposes animals to a spectrum of co-morbidities, such as orthopedic disease, insulin resistance, cardiovascular dysfunction, cancer, and steatotic liver disease (1, 2). Obesity has been linked to reduced lifespan, with obese dogs living up to 1.5 years less than their lean counterparts (1). Moreover, the financial and psychological burden of managing obesity-related diseases in pets can be substantial, disproportionately affecting households with lower socioeconomic status (3).

Current large-scale epidemiological studies have highlighted that the risk of being overweight or obese varies considerably by life stage, breed, and reproductive status (1, 4). Recent findings in Labrador retrievers have identified DENND1B as a key obesity-associated gene that modulates melanocortin 4 receptor (MC4R) signaling, a pathway central to appetite regulation in both dogs and humans. The same gene is also linked to body mass index in large human cohorts and severe childhood obesity, highlighting a shared genetic basis across species, positioning the dog as a powerful translational model (5). These genetic predispositions, combined with lifestyle factors such as *ad libitum* feeding and physical inactivity, help explain the rising prevalence of obesity even in juvenile life stages (1).

While obesity in dogs has been associated with systemic inflammation, insulin resistance, dyslipidemia, and alterations in gut microbiota composition (2, 6, 7), the mechanistic understanding of these processes in companion animals remains limited. In dogs, high-fat diets have been linked to colonic microbiota deviations characterized by Proteobacteria overgrowth, mucosal inflammation, and increased epithelial apoptosis, suggesting early disruption of gut immune homeostasis. However, pathophysiological mechanisms underlying obesity and its comorbidities have been extensively delineated in humans and rodent models but not in companion animals. Microbiota deviations combined with disruption of gut barrier integrity lead to microbial translocation, particularly lipopolysaccharide (LPS), and activation of pro-inflammatory signaling cascades (e.g., TLR4 - NF-κB) that drive systemic inflammation and hepatic dysfunction (8). Bile acid dysregulation has emerged as a central driver of metabolic disease, with altered bile acid pools and impaired FXR and TGR5 signaling linked to increased intestinal permeability, dysregulated lipid and glucose metabolism, reduced GLP-1 secretion, and exacerbation of chronic inflammation (9). Whether such an integrated gut–liver–metabolic axis involving bile acid signaling operates similarly in dogs remains to be established.

Despite these insights, current management of obesity in companion animals remains largely reliant on caloric restriction and increased physical activity. These approaches are often difficult to sustain in practice and can bring along significant health risks in cats, as rapid weight loss can induce feline hepatic lipidosis (10). Recent insights into host–microbiota interactions have repositioned the gastrointestinal tract as a key target for dietary modulation, offering new perspectives on nutritional strategies to support metabolic health in the context of obesity. Among microbial candidates, supplementation of *Akkermansia muciniphila* Muc^T^ has emerged as a particularly promising approach, with consistent evidence from preclinical and clinical studies showing the capacity of this strain to enhance gut barrier function, modulate immune tone, and improve metabolic health (11). While certain live probiotics have demonstrated beneficial effects on lipid metabolism and inflammation (12) in canines, their application remains constrained by challenges related to viability, formulation, and shelf-life. Notably, both live and pasteurized *Akkermansia muciniphila* Muc^T^ have previously been shown to improve metabolic health in rodents and humans, with some even reporting greater efficacy of the pasteurized form (13–15). This observation has been linked to the preservation of bioactive structural components, including the abundant outer membrane protein Amuc_1100, a thermostable protein implicated in host immune and metabolic signaling through Toll-like receptor 2 dependent pathways (14). As a strict anaerobe, formulation and administration of live *A. muciniphila* Muc^T^ remain technically challenging, whereas pasteurization improves stability, shelf-life, and compatibility with a wide range of feed applications while retaining biological activity. In contrast, due to the thermal stability of the abundant outer membrane protein Amuc_1100, which is considered an important mediator of its mode of action, pasteurized *A. muciniphila* Muc^T^ retains functional activity while offering improved stability and broader applicability across feed formats, as shown in rodents and humans (13–15).

Given the chronic trajectory and economic burden of obesity in pets, there is an urgent need for gut-targeting strategies that not only reduce excessive weight but also address obesity-associated mucosal barrier dysfunction, chronic inflammation, and hepatic injury. While the metabolic and immunomodulatory benefits of pasteurized *A. muciniphila* Muc^T^ have been demonstrated in rodents and humans, evidence in companion animals remains scarce. Therefore, we performed a proof-of-concept study directly in dogs to evaluate whether the previously reported immune-metabolic benefits of pasteurized *Akkermansia muciniphila* Muc^T^ are also observed during high-caloric diet-induced weight gain, with a particular focus on body weight development, systemic inflammation, and gut–liver–immune axis modulation.

## RESULTS

### Pasteurized *Akkermansia muciniphila* Muc^T^ reduces weight gain during high-caloric diet feeding

To assess the anti-obesity and anti-inflammatory potential of pasteurized *Akkermansia muciniphila* Muc^T^, Beagle dogs were fed a high-caloric, high-fat, and high-protein commercial diet (Inukshuk 32/32) for 8 weeks (here denoted as high-caloric diet). The study population consisted of 20 healthy Beagle dogs (six males and 14 females), randomized into two groups balanced for sex, body weight, body condition score, and age. The treatment group (*n* = 10; three males, seven females) received 3 × 10⁹ total fluorescent units (TFU) per day of pasteurized *A. muciniphila* Muc^T^, administered daily by mixing with a standard wet diet, whereas the control group (*n* = 10) received only an unamended standard wet diet (Cesar Classic Loaf in Sauce Filet Mignon; here denoted as the control diet).

As expected, the high-caloric diet induced progressive weight gain in all animals, except for one control dog that displayed a near to zero feed conversion ratio, despite adequate caloric intake. Such resistance to diet-induced weight gain has been recognized in rodent models and is often attributed to inherent metabolic differences (16, 17). In addition, one treatment dog consistently refused the wet diet and thus was not exposed to the intervention. Both animals were excluded from the analyses, resulting in *n* = 9 per group.

**Fig 1:**
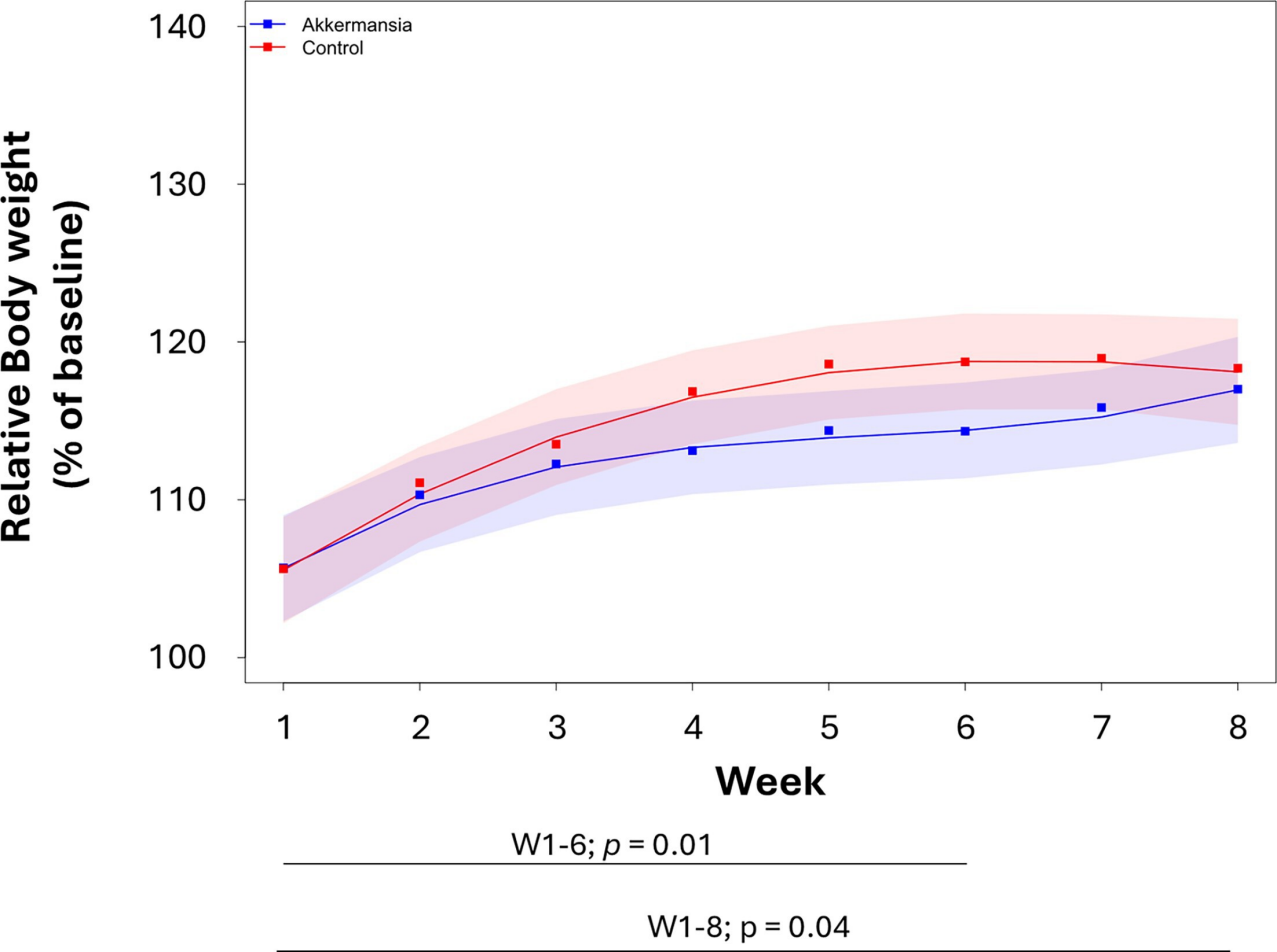
*Akkermansia muciniphila* Muc^T^ supplementation attenuated body weight gain in dogs exposed to a high-caloric diet. Body weight was measured weekly throughout the 56-day intervention and expressed as relative body weight (% of baseline body weight at week 1) to normalize for inter-individual differences in starting body weight and facilitate comparison of weight gain trajectories across animals. Filled squares represent the observed weekly group means for dogs receiving pasteurized *A. muciniphila* Muc^T^ (blue) or placebo control (red). Solid lines represent model-estimated mean body weight trajectories derived from a linear mixed-effects model (LMM) including treatment group, sex, a third-order polynomial term for time (week), and random intercepts for individual dogs to account for repeated measurements. Shaded regions indicate the corresponding 95% confidence intervals around the model predictions. Statistical significance was assessed using the full longitudinal LMM and therefore reflects differences in overall weight gain trajectories rather than comparisons at individual weeks. The treatment effect was significant over both weeks 1–6 (*P* = 0.01) and the complete intervention period of weeks 1–8 (*P* = 0.04), indicating a sustained attenuation of diet-induced weight gain by pasteurized *A. muciniphila* MucT.

*A. muciniphila* Muc^T^ supplementation significantly reduced the overall weight gain trajectory compared to the control diet during the period in which voluntary food intake was comparable between groups (week 1–6; Linear Mixed Model (LMM): time × treatment interaction: *P* = 0.01, Fig. 1). To account for inter-individual differences in baseline body weight, including those associated with gender, body weight trajectories were analyzed and visualized as relative body weight (% of each dog’s baseline body weight at study initiation). After 6 weeks, mean body weight had increased by 14.3 ± 4.8% in the pasteurized *A. muciniphila* group compared to 18.7 ± 6.5% in controls, corresponding to the period of greatest numerical separation between groups within the significant longitudinal time × treatment interaction model.

When extending the analyses to the full intervention period (weeks 1–8), which included increased variability in voluntary food intake and diet refusal in several control animals during the final 2 weeks, the treatment effect on body weight gain remained statistically significant, albeit attenuated (LMM: time × treatment interaction: *P* = 0.04, Fig. 1). These *P* values reflect differences in the overall longitudinal body weight trajectories between groups rather than comparisons at individual weekly time points. On an absolute scale, this resulted in a smaller difference in mean body weight gain between the two treatment groups by the end of the clinical study, reflecting partial convergence of weight trajectories during a late phase characterized by altered feeding behavior in control animals.

Voluntary food intake did not differ significantly between treatment groups when analyses using the same LMM statistics applied to body weight gain. However, from week 7 onward, half of the animals in the control group exhibited sustained reductions in food intake, coinciding with body weight loss. This late-phase pattern was not observed in the *Akkermansia* treatment group. Accordingly, voluntary food-intake data were interpreted descriptively and considered supportive rather than determinative for the observed body weight trajectories.

During the clinical trial, body condition score (BCS) values were assessed weekly by trained personnel using a standardized 5-point scale varying from thin to obese (1 to 5, respectively). Violin plots (Fig. S1) showed a distribution at 8 weeks skewed toward higher BCS values in the control group, while dogs receiving *A. muciniphila* Muc^T^ exhibited lower median scores (NS).

### A high-caloric diet alters systemic markers of metabolism, inflammation, and liver function: pasteurized *A. muciniphila* Muc^T^ reduces systemic and gastrointestinal inflammation

**Fig 2:**
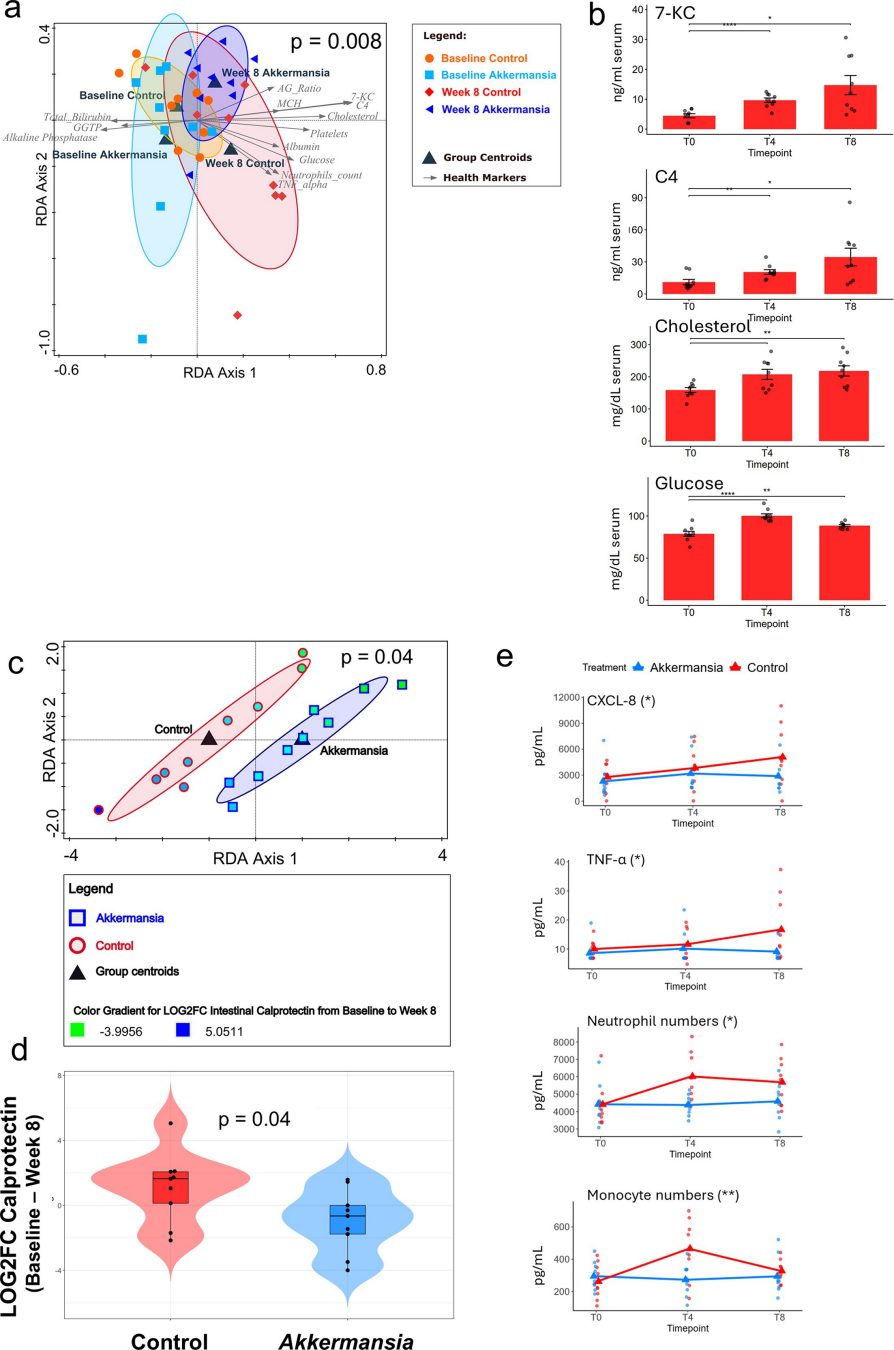
A high-caloric diet induces systemic inflammation, hyperlipidemia, and liver stress, where *Akkermansia muciniphila* Muc^T^ treatment specifically affects systemic and gastrointestinal inflammation. (**a**) High-caloric diet intake led to a significant shift in the overall health phenotype between baseline and week 8 (*P* = 0.008). This was demonstrated by redundancy analysis (RDA) with metabolic, inflammatory, and hematological markers as response variables and a combination of time point and treatment as explanatory variables. The shift was primarily driven by increased levels of the following markers (arrows): bile acid synthesis rate markers 7-ketocholesterol (7-KC) and 7α-hydroxy-4-cholesten-3-one (C4), cholesterol, glucose, platelet counts, mean corpuscular hemoglobin (MCH), albumin, neutrophils, albumin-to-globulin ratio, TNF-α, and total white blood cells. Large triangles (black) are centroids of the sample groups (Time × Treatment), while the other symbols indicate individual samples. Ellipses are the 66% quantile of the approximated 2D-normal density distribution function for each sample group. (**b**) Longitudinal serum biomarker analysis of control-treated animals fed a high-caloric diet. Bar graphs depict mean ± SEM concentrations of 7-ketocholesterol (7-KC), 7α-hydroxy-4-cholesten-3-one (C4), glucose, and total cholesterol at baseline (T0), week 4 (T4), and week 8 (T8). Individual data points represent measurements from single animals. Statistical comparisons were performed using paired within-subject analyses comparing T4 and T8 to baseline. Significance levels are indicated above the bars (*P < 0.05; **P < 0.01; ***P < 0.001; ****P < 0.0001; ns, not significant). These analyses demonstrate a progressive deterioration of lipid and glucose homeostasis in control animals during high-caloric feeding. (**c**) *Akkermansia muciniphila* Muc^T^ treatment significantly altered systemic health parameters (*P* = 0.04), with treated samples showing reduced markers of systemic and gastrointestinal inflammation. RDA was performed on log₂ fold changes of week 8 versus baseline samples of all collected systemic health parameters, with treatment as explanatory variable. Key contributing variables included calprotectin, TNF-α, monocyte and neutrophil counts, globulin, white blood cell count, C-reactive protein, albumin, and interleukin-8. (**d**) Log₂ fold changes from baseline to week 8 in fecal calprotectin levels were dampened in the *A. muciniphila* Muc^T^–treated group compared to controls, as confirmed by the attribute plot where calprotectin levels are driving treatment separation in the RDA. (**e**) *A. muciniphila* Muc^T^ supplementation ameliorated diet-induced systemic immune activation. Treatment × time interaction testing revealed significantly reduced plasma levels of the pro-inflammatory cytokine TNF-α, the chemokine CXCL-8, and decreased circulating numbers of monocytes and neutrophils in the treatment group compared to controls. Plots show individual data points and model-based estimated marginal means from LMM analyses (connected with lines representing treatment groups). Statistical significance is indicated as: *P* < 0.05 (*), *P* < 0.01 (**), *P* < 0.001 (***), *P* < 0.0001 (****).

High-fat diet feeding is known to induce systemic metabolic stress, liver dysfunction, and low-grade inflammation in both humans and animals. To capture these effects, redundancy analysis (RDA) was performed with time point and treatment as explanatory variables and a panel of metabolic, hematological, and inflammatory markers as response variables, including indicators of bile acid synthesis rate from cholesterol such as 7-ketocholesterol (7-KC) and 7α-hydroxy-4-cholesten-3-one (C4). A significant shift in the combined health phenotype was observed from baseline to week 8 (*P* = 0.008; Fig. 2a). This phenotype was primarily characterized by, ranked in order of importance, increases in 7-KC, C4, cholesterol, platelet counts, glucose, mean corpuscular hemoglobin (MCH), albumin, neutrophil counts, the albumin-to-globulin ratio, TNF-α, and total white blood cell counts. These diet-induced alterations were further confirmed by longitudinal within-subject analyses restricted to control animals, which revealed significant increases over time for the parameters 7-KC, C4, cholesterol, and glucose (Fig. 2b).

To evaluate whether supplementation with pasteurized *A. muciniphila* Muc^T^ modulated systemic responses to the high-caloric diet, a second RDA was performed on the log₂ fold change (T8 vs T0) of all measured health parameters comparing treatments. The ordination revealed a significant association between health parameters and treatment (*P* = 0.04; Fig. 2c). Samples from the *A. muciniphila* Muc^T^–treated group clustered separately, primarily driven by reductions in markers of systemic inflammation. In order of contribution, the most strongly affected variables were fecal calprotectin, TNF-α, absolute monocyte and neutrophil counts, globulin levels, white blood cell counts, CRP, albumin, and CXCL8. The strongest driver of the treatment separation was calprotectin, as confirmed by attribute plots and univariate testing, with significantly lower log₂ fold changes in the treatment group (Fig. 2d). Longitudinal mixed-effects modeling identified significant time × treatment interactions for circulating CXCL8, TNF-α, monocytes, and neutrophils (Fig. 2e), demonstrating that the temporal response profiles of these markers differed between treatment groups over the course of the intervention, consistent with the multivariate treatment-associated shift identified by the RDA.

### High-caloric diet alters fecal lipid, bile acid, and microbiota composition: *Akkermansia muciniphila* Muc^T^ impacts specific taxa only

A high-protein, high-fat animal-based diet in humans rapidly restructures the gut microbiota by enriching taxa associated with inflammation and depleting fiber-fermenting *Firmicutes*, thereby reducing SCFA production and shifting microbial metabolism toward protein utilization (18). These diet-induced alterations in microbial composition can, in turn, modulate bile acid composition and transformation. To assess dietary effects and the potential modulation by pasteurized *A. muciniphila* Muc^T^, fecal samples were analyzed for lipid and bile acid profiles using GC-MS and LC-MS/MS, and microbiota composition was profiled via 16S rRNA gene amplicon sequencing and qPCR.

Redundancy analysis revealed a significant shift in fecal fatty acid composition following 8 weeks of high-caloric diet feeding (*P* = 0.002, Fig. S2A). This shift was characterized by a reduction in plant-derived sterols (campesterol, fucosterol, stigmasterol, and β-sitosterol) and polyunsaturated fatty acids (linoleic acid and alpha-linolenic acid). In contrast, levels of cholesterol and its microbial conversion products (cholestanol and coprostanol) increased, alongside saturated long-chain fatty acids (myristic acid [C14:0] and stearic acid [C18:0]) and nervonic acid, a marker of gut mucosal damage. These changes suggest a diet-induced transition toward higher intestinal lipid load and altered microbial sterol metabolism. Multivariate analyses showed no significant treatment effect of pasteurized *A. muciniphila* Muc^T^ supplementation on the fecal lipidome, neither at week 8 nor based on log₂ fold changes from baseline.

**Fig 3:**
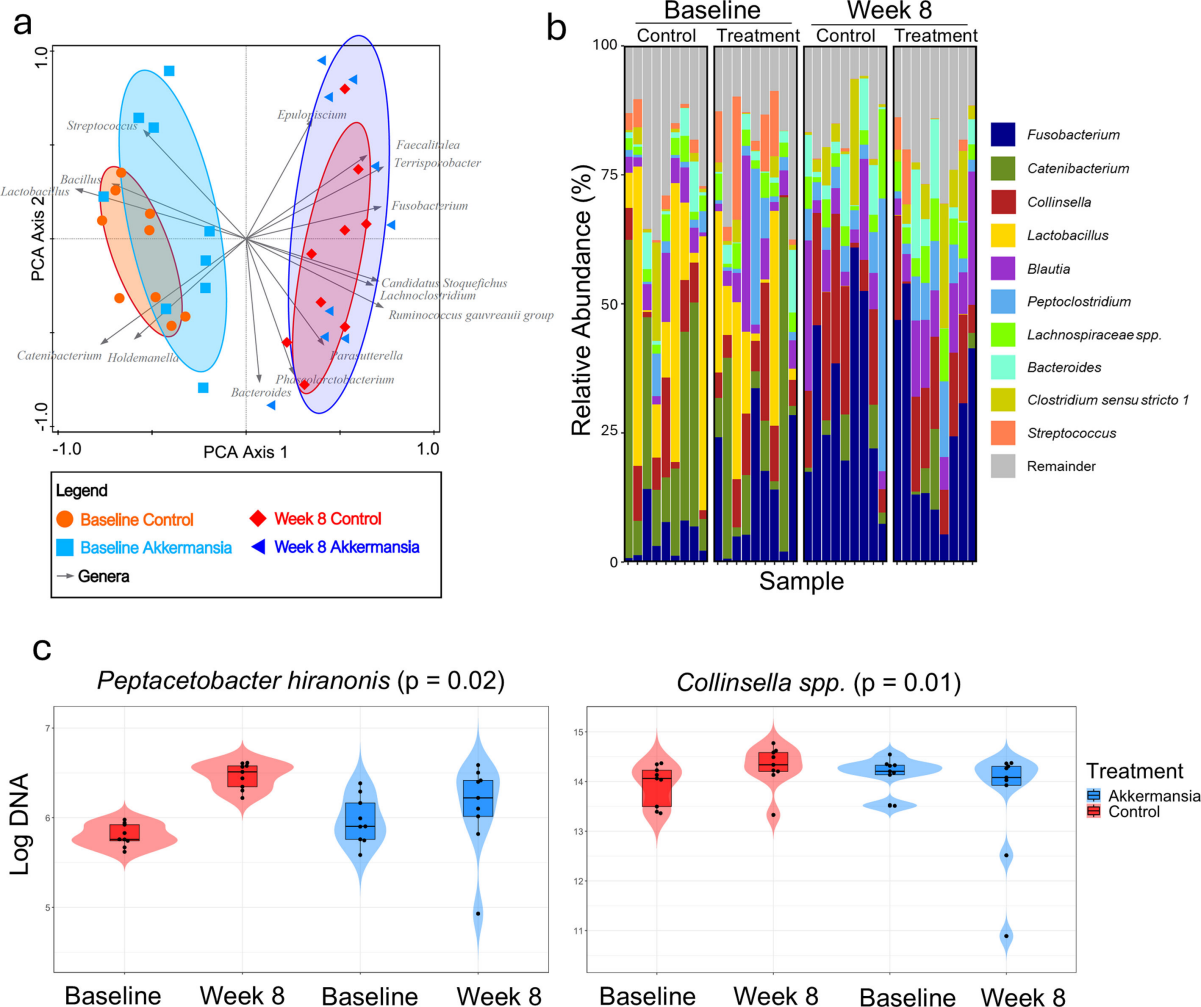
High-caloric diet alters microbiota composition; only specific taxa are modulated by *Akkermansia muciniphila* Muc^T^ treatment. (**a**) Principal component analysis (PCA) of genus-level relative abundances reveals a clear shift in microbial community composition in response to high-caloric diet feeding. Ellipses are the 66% quantile of the approximated 2D-normal density distribution function for each sample group. Gray arrows are the 15 best-fitting genera. (**b**) Stacked bar plot showing the relative abundance of dominant microbial genera across sample groups defined by time point (baseline and week 8) and treatment (control vs *A. muciniphila* Muc^T^). The plot highlights diet-induced changes, including an increase in *Fusobacterium* and members of the Clostridiales order, and a decrease in carbohydrate-fermenting *Firmicutes*. Each bar represents the average taxonomic composition of samples for each individual sample grouped by time point and treatment, respectively. Taxa are aggregated at the genus level, color-coded by taxonomic identity, and limited to the top 10 most abundant genera; remaining genera are grouped under “Remainder.” (**c**) Quantitative PCR (qPCR) of selected taxa confirms the compositional shifts observed in sequencing data. *Peptacetobacter hiranonis* and *Collinsella* spp. abundance increased significantly in response to high-caloric diet feeding, and these increases were dampened by the *A. muciniphila* Muc^T^ treatment during the 8-week period. Data are expressed as Tukey-style boxplots of log DNA abundance and individual data points overlaid. Statistical significance was assessed using Wilcoxon rank-sum tests comparing log₂ fold changes between treatment groups.

Sequencing of the amplicons of the 16S rRNA V4 region revealed a total of 1,941 amplicon sequence variants (ASVs), of which 154 were assigned to a specific genus at ≥97% confidence using the SILVA database, which contains the most complete catalogs of environmental sequences (v138). Alpha diversity indices, including observed features, Pielou’s evenness, Shannon entropy, and Faith’s phylogenetic diversity, were not significantly affected by the high-caloric diet nor the pasteurized *A. muciniphila* Muc^T^ treatment (Fig. S3). However, principal component analysis (PCA) of genus-level relative abundance revealed a pronounced shift in microbiota composition in response to the high-caloric diet (Fig. 3a). This shift was characterized by a reduction in the relative abundance of several *Firmicutes* genera involved in carbohydrate fermentation (*Lactobacillus*, *Catenibacterium*, *Bacillus*, and *Streptococcus*) and a concurrent increase in *Fusobacterium* and members of the Clostridiales order (Fig. 3b). Subsequent qPCR analysis confirmed these trends for selected taxa, with a significant increase in *Peptacetobacter hiranonis* (previously known as *Clostridium hiranonis*), *Fusobacterium,* and *Collinsella* spp., accompanied by decreased *Prevotella copri*, *Lactobacillus*, and *Streptococcus* spp., as evidenced by their log DNA levels (Fig. S4). These shifts, however, remained mostly within the reference intervals established for healthy dogs, and, consequently, did not affect the score in the earlier developed Canine Dysbiosis Index (19, 20). While no significant treatment effects were detected in 16S rRNA-based microbiota profiles, targeted qPCR revealed that pasteurized *A. muciniphila* Muc^T^ supplementation significantly reduced the high-caloric diet-induced log2 fold increases in *P. hiranonis* and *Collinsella* spp. relative to the control group (Fig. 3c).

Fecal bile acid profiling showed significant alterations induced by the high-caloric diet (*P* = 0.004), characterized by increased levels of secondary, conjugated, and oxidized bile acids, which indicate increased bile acid production induced by the high-fat content of the diet, increased levels of bile acid–metabolizing bacterial taxa, and altered bile acid recycling (Fig. S2B). Notably, redundancy analysis indicated that *P. hiranonis*, a key 7α-dehydroxylating bacterium responsible for converting primary to secondary bile acids in dogs, was strongly associated with the fecal bile acid profile (*P* = 0.002; 16.8% explained variation; Fig. S5a and b). Its abundance correlated positively with levels of lithocholic acid (LCA), deoxycholic acid (DCA), and its oxidized derivatives (12-oxo-LCA, 3-oxo-DCA), as well as with markers of active hepatic detoxification (taurine-conjugated bile acids, including TUDCA, TDCA, TCDCA, and TLCA), supporting a role for this taxon in shaping the secondary bile acid pool under dietary lipid stress.

### Microbiota-inflammation interactions may underlie appetite suppression during late-stage weight gain reduction

To investigate whether microbiota composition correlated with inflammatory status and appetite suppression, further redundancy analysis was performed integrating microbiota fold changes (T0–T8), food intake, and inflammatory markers (Fig. S6). This analysis revealed a significant association between microbial composition and food intake at week 8 (*P* = 0.028). Lower food intake was associated with increased relative abundance of proteolytic taxa such as *Fusobacterium* and *Clostridium sensu stricto* 13 and reduced relative levels of commensals such as *Lactobacillus* and *Ruminococcus* spp. Notably, *Fusobacterium* abundance, measured by qPCR, aligned with 16S rRNA-derived *Fusobacterium* data, indicating consistent patterns across the used methodologies. The observed microbiota shifts aligned with inflammatory markers and reduced food intake, suggesting that diet-induced shifts and systemic inflammation may have contributed to appetite suppression. This host response of reduced food intake likely underlies the reduced weight gain in control animals during the final phase of the intervention, leading to the partial convergence in weight trajectories (Fig. 1)

### Multi-omics factor analysis reveals alleviation of diet-induced host-microbial dysregulation by pasteurized *A. muciniphila* Muc^T^

**Fig 4:**
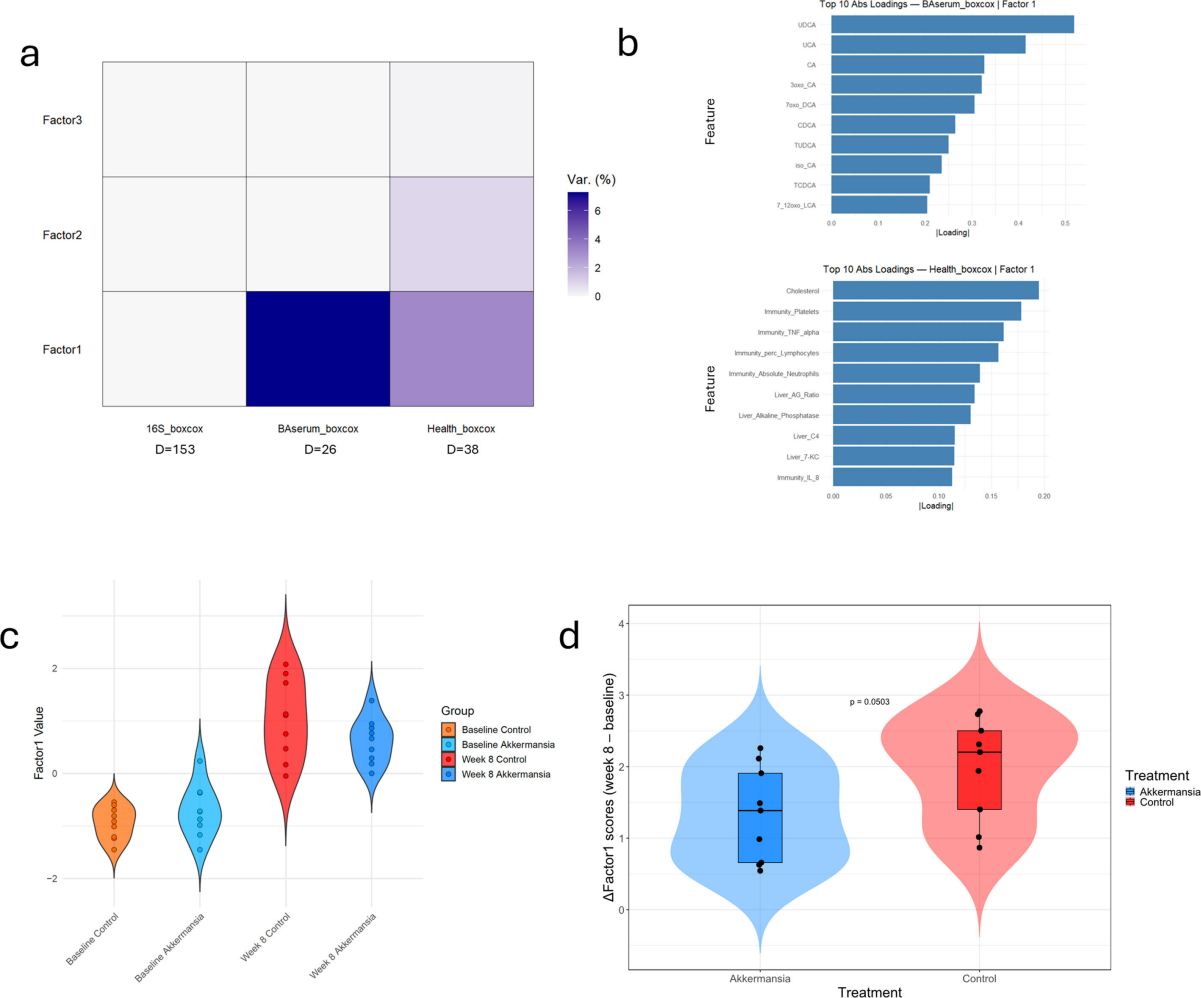
Multi-omics factor analysis (MOFA) reveals alleviation of diet-induced host-microbial dysregulation by *Akkermansia muciniphila* Muc^T^ treatment. (**a**) Study overview and data types included in the MOFA are shown in different rows (*D* = number of features) with the proportion of total variance explained (%) by individual factors shown for each data set in the heatmap. Variance decomposition of Factor 1 shows that serum bile acids contributed the largest share of explained variance, followed by systemic host health markers, and fecal microbiota composition contributed minimally. (**b**) Top features driving Factor 1 include primary bile acids, their secondary and oxidized derivatives, and elevated plasma cholesterol, oxidized cholesterol (7-KC), bile acid synthesis marker C4, pro-inflammatory cytokines (TNF-α, IL-8/CXCL-8), neutrophil counts, and liver stress markers (albumin-globulin ratio and alkaline phosphatase). (**c**) A coordinated shift in Factor 1 is observed in the control group following high-caloric diet feeding, while this shift is significantly attenuated in the *A. muciniphila* Muc^T^–treated group (*P* = 0.05, **d**), suggesting dampened gut–liver–immune system perturbations. Together, these features reflect a diet-induced axis of bile acid metabolism, systemic inflammation, and lipid dysregulation, moderated by *A. muciniphila* Muc^T^ supplementation. ΔFactor 1 scores are shown as violin plots with Tukey-style boxplots and jittered data points (control = red, *Akkermansia* = blue). Statistical differences were tested with the Wilcoxon rank-sum test.

To better understand how pasteurized *A. muciniphila* Muc^T^ would modulate the interplay between gut microbiota, microbial metabolites, and host health under high-caloric dietary pressure, we performed a multi-omics factor analysis (MOFA). MOFA is a statistical framework that generalizes principal component analysis (PCA) for multi-modal biological data sets to identify latent factors that capture shared sources of variability across samples. Here, three omics layers were analyzed jointly, namely fecal microbiota composition (16S metagenomics), serum bile acid profiles (metabolomics), and systemic host health markers (inflammatory, metabolic, and hematological parameters) collected from the same animals (Fig. 4a). The first latent factor (Factor 1) captured the dominant source of variance across the combined data sets (Fig. 4b). Visualization of individual Factor 1 scores revealed a coordinated shift in multi-omics profiles induced by high-caloric feeding, as evidenced in the control group (Fig. 4c). This shift was significantly reduced in animals treated with pasteurized *A. muciniphila* Muc^T^ (*P* = 0.05), indicating a dampening of high-caloric diet-induced gut–liver–immune system alterations (Fig. 4d).

The variance decomposition across omics layers showed that serum bile acids contributed the largest proportion of explained variance to Factor 1, followed by systemic host health markers. Fecal microbiota composition contributed minimally to this factor. The top features driving this latent factor (Fig. 4b) included several bile acid species, notably the primary bile acid cholic acid (CA) and chenodeoxycholic acid (CDCA), which are synthesized in the liver from cholesterol, as well as their secondary and oxidized derivatives, including ursodeoxycholic acid (UDCA), ursocholic acid (UCA), 7-oxo-DCA, and 3-oxo-CA. These were accompanied by elevated plasma levels of cholesterol, 7-KC (an oxidized form of cholesterol), C4 (a bile acid synthesis marker), the pro-inflammatory cytokines TNF-α and CXCL8 as well as neutrophil count, and the liver markers albumin-globulin and alkaline phosphatase (Fig. 4b). To facilitate interpretation of Factor 1, the longitudinal profiles of the highest-weighted bile acid, metabolic, and immune-related features are presented in Fig. S7, illustrating the coordinated multivariate patterns captured by the MOFA and providing context for features that were significant in targeted univariate analyses (Fig. 2; Fig. S8). Together, these results suggest that MOFA captured a coherent, diet-induced physiological axis defined by altered bile acid metabolism, systemic inflammation, and lipid (cholesterol) dysregulation that was significantly moderated by pasteurized *A. muciniphila* Muc^T^ supplementation. Notably, this latent factor comprised both variables that were individually associated with *A. muciniphila* Muc^T^ supplementation and variables that primarily contributed through their covariance with other features, illustrating the ability of MOFA to capture coordinated biological responses that extend beyond univariate statistical testing.

### Pasteurized *A. muciniphila* Muc^T^ reduces circulating secondary bile acids and associated inflammatory signatures

**Fig 5:**
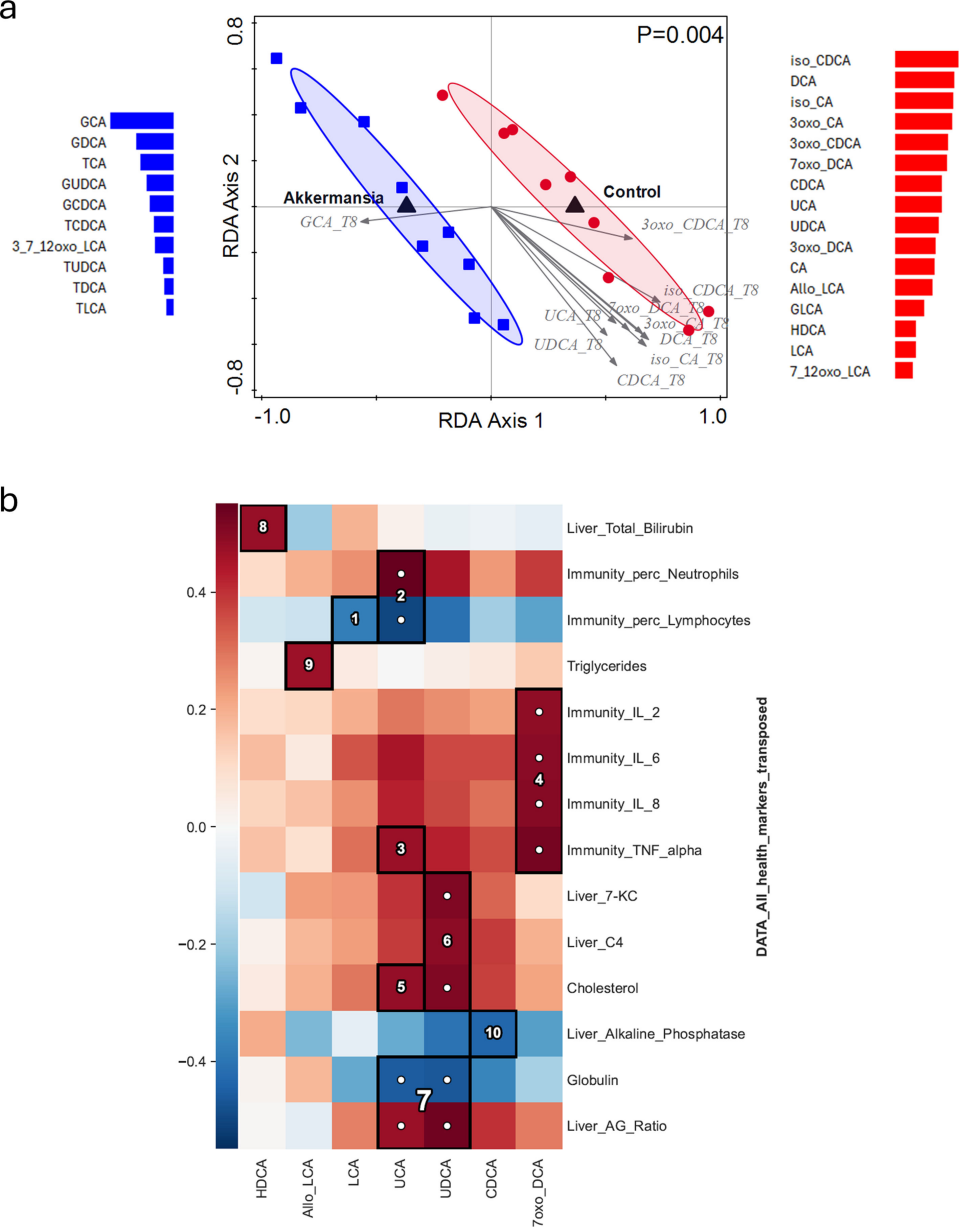
*Akkermansia muciniphila* Muc^T^ reduces systemic accumulation of secondary and microbially modified primary bile acids linked to inflammation. (**a**) Supplementation with *A. muciniphila* Muc^T^ prevented the systemic build-up of microbially modified bile acid derivatives (*P* = 0.004), which are markers of impaired hepatic repair capacity and oxidative stress. Redundancy analysis of log₂ fold changes in serum bile acids (weeks 0–8) was performed with time point × treatment as explanatory variables. Symbols indicate individual samples, colored by treatment group. Ellipses are the 66% quantile of the approximated 2D-normal density distribution function for each treatment. Gray arrows are the 12 best-fitting bile acids. (**b**) Hierarchical all-against-all association (HAllA) revealed that variation in bile acid composition was significantly associated with systemic health parameters, with strongest associations observed for 7-oxo-DCA levels, which were positively associated with systemic inflammation. Heatmaps were generated using HAllA (v0.8.40) with Spearman correlation, hierarchical clustering of features in both data sets, and false discovery rate control at α = 0.1 based on permutation testing. Rows represent health markers and columns serum bile acids (selection as identified by the RDA for the control group) (**a**), ordered by hierarchical clustering. Color intensity reflects correlation strength (red = positive, blue = negative), while statistically significant blocks of associations identified by HAllA are delineated by rectangles. In the corresponding hallagram, the numbers within blocks denote the rank order of significance, with 1 representing the block with the lowest FDR-adjusted *P* value.

To further explore the importance of serum bile acid profiles as identified by MOFA, we performed redundancy analysis to assess the impact of pasteurized *A. muciniphila* Muc^T^ supplementation on log₂ fold changes in serum bile acids from baseline to week 8. The supplementation significantly reduced the systemic accumulation of microbially modified bile acids (Fig. 5a). The most prominent divergence between the control and pasteurized *A. muciniphila* Muc^T^–treated animals was a striking increase in oxidized forms of cholic acid (CA), chenodeoxycholic acid (CDCA), and lithocholic acid (LCA) in the control group (log₂ FC ~2–3), which was absent in the *A. muciniphila* Muc^T^ treatment group (log₂ FC ~0) (Fig. S7). These oxidized derivatives are markers of impaired hepatic repair capacity and oxidative stress, both of which exacerbate systemic and hepatic inflammation (21), which was confirmed by hierarchical all-against-all association (HAllA) analysis, where 7-oxo-DCA levels positively correlated with serum inflammatory cytokine levels, including TNF-α and CXCL8 (Fig. 5b). Consistent with the serum profiles, the high-caloric diet induced a shift in fecal bile acids primarily characterized by microbially modified species (Fig. S2B). These were positively associated with bile acid–modifying taxa such as *Fusobacterium*, *P. hiranonis*, *C. perfringens*, and *E. coli*, and negatively correlating with beneficial short-chain fatty acid producers such as *Ruminococcus gnavus*, *Faecalibacterium*, and *Blautia* (Fig. S5B). Notably, pasteurized *A. muciniphila* Muc^T^ supplementation did not directly alter fecal bile acid composition, indicating that the observed treatment effects were not reflected at the level of luminal bile acid pools. As many secondary bile acid species, particularly oxidized and unconjugated forms, undergo modification upon enterohepatic circulation, the systemic accumulation patterns observed here are compatible with altered host handling of bile acids. However, the underlying processes cannot be inferred without direct hepatic measurements.

Collectively, these findings suggest that pasteurized *A. muciniphila* Muc^T^ supplementation effectively attenuates high-caloric diet-induced systemic inflammation, thereby potentially alleviating hepatic stress resulting in more effective clearance of harmful bile acid species.

## DISCUSSION

*Akkermansia muciniphila* is a prominent mucus-degrading bacterium of the gastrointestinal microbiota. Since its discovery two decades ago, low or decreasing abundance of *A. muciniphila* has been linked with several diseases such as obesity, diabetes, liver steatosis, and inflammation (11). Its abundance was significantly reduced in individuals with obesity and metabolic syndrome and was negatively affected by high-fat diet feeding in both humans and animals (11, 22). The therapeutic potential of the best-studied strain, *A. muciniphila* Muc^T^, has been established through oral supplementation studies in which both live and, even more effectively, pasteurized forms of the bacterium mitigated various features of metabolic disease (11). In mice and humans, both live and pasteurized *A. muciniphila* Muc^T^ supplementation reduced fat mass gain, improved insulin sensitivity, and attenuated dyslipidemia, including reductions in serum cholesterol and triglyceride levels (13, 14). In prior mechanistic studies, pasteurized *A. muciniphila* Muc^T^ was found to protect the intestinal barrier and reduce gut inflammation, partly through the action of its thermostable and abundant outer membrane protein Amuc_1100, which interacts with host Toll-like receptor 2 (TLR2) (14, 23). These immune-metabolic interactions are not confined to preclinical models, as human trials confirmed the safety and metabolic benefits of pasteurized *A. muciniphila* Muc^T^ supplementation, including improvements in insulin sensitivity and circulating lipid profiles (13).

Two earlier studies investigated the effects of live or heat-treated A. muciniphila preparations in dogs fed a high-fat diet (24, 25). However, these studies used different strains and employed processing methods that differed substantially from the standardized pasteurization procedure used for *A. muciniphila* Muc^T^ in the present study. Importantly, the pasteurization protocol applied here was specifically optimized to preserve bioactive structural components and signaling molecules associated with the proposed mechanism of action, including the outer membrane protein Amuc_1100, while ensuring loss of viability. This differs from more prolonged heat treatment procedures described by Lin et al. (24), which may result in partial denaturation of heat-sensitive bioactive components. Both studies showed significant reductions in body weight gain and varying improvements of blood glucose, triglycerides, and cholesterol. Our study evaluated the effects of pasteurized *A. muciniphila* Muc^T^ supplementation during consumption of a commercially available high-caloric diet designed to induce energy excess through overfeeding, a common contributing factor to weight gain in companion dogs (7). Dogs with a healthy baseline body condition received a diet enriched in lipids and proteins at approximately three times their daily energy requirements, thereby creating a nutritional context associated with weight gain and metabolic stress under high-caloric feeding conditions. To delineate biological pathways associated with the observed effects beyond body weight regulation in canines, we conducted a comprehensive multi-omics analysis of gut microbiota, systemic and intestinal inflammatory markers, liver markers, and fecal and serum lipid and bile acid profiles. While the present study was not designed as a dedicated mechanistic investigation, integration of these data sets enabled exploration of biological processes previously implicated in the mode of action of pasteurized *A. muciniphila* Muc^T^ in rodents and humans.

Control animals receiving the high-caloric diet displayed rapid and consistent weight gain, ranging from 11.3% to 23.4% (average 15.2%) over the 8-week intervention period. In contrast to commonly used high-fat-only experimental diets, the commercially available diet used in this study contained elevated levels of both lipids and proteins. Consequently, weight gain likely reflected a combination of increased adiposity and lean mass accretion, potentially reducing sensitivity for detecting body weight–specific intervention effects of pasteurized *A. muciniphila* Muc^T^.

In addition, several control animals showed reduced voluntary feed intake during the later phase of the intervention. This reduction coincided with stronger microbiota alterations, elevated inflammatory markers, and increased fecal calprotectin levels, suggestive of intestinal distress and disturbed homeostasis rather than physiological satiety alone. These effects were markedly less pronounced in animals receiving pasteurized *A. muciniphila* Muc^T^.

The high-caloric diet induced profound and congruent changes in the intestinal compartment in both treatment and control groups, characterized by increased and distorted fecal lipid profile, a compensatory bile acid response, and a profound change in microbiota composition. The high-caloric diet reduced levels of anti-inflammatory polyunsaturated fatty acids such as linoleic acid and cholesterol-lowering phytosterols, including stigmasterol and β-sitosterol, lipid species previously associated with improved metabolic outcomes and reduced inflammatory tone (26). In parallel, increased levels of cholesterol and its microbial metabolites (cholestanol, coprostanol), several saturated long-chain fatty acids, and nervonic acid, a lipid marker linked to mucosal injury, were observed (27). These findings reflect a shift towards excessive luminal lipid burden and epithelial stress, in line with previous studies demonstrating increased colonic permeability under high-fat dietary conditions in dogs (28).

This lipid accumulation was accompanied by a distinct remodeling of the bile acid pool. Fecal bile acid profiling revealed increased concentrations of secondary, taurine-conjugated, and oxidized bile acids, indicating enhanced hepatic bile secretion, intensified microbial bile acid transformation, and impaired enterohepatic recycling (29). These alterations are consistent with known high-fat diet-induced upregulation of hepatic bile acid synthesis, which potentially favors growth of bile acid–metabolizing bacteria (30). Redundancy analysis identified *Peptacetobacter hiranonis*, a key 7α-dehydroxylating bacterium in dogs (31), as a primary driver of this shift, with strong positive correlations to lithocholic acid (LCA), deoxycholic acid (DCA), and its oxidized derivatives, including 12-oxo-LCA and 3-oxo-DCA, derived from the concerted hydroxysteroid dehydrogenase (HSDH) activity by *P. hiranonis* and other microbial community members (32).

In parallel, consumption of the high-caloric diet was associated with functionally relevant shifts in fecal microbiota composition relative to baseline. Although overall alpha diversity and the Canine Dysbiosis Index remained largely unaffected, reductions in saccharolytic Firmicutes genera, including Lactobacillus, *Catenibacterium, Bacillus*, and *Streptococcus*, may reflect the relatively low carbohydrate content of the protein- and lipid-enriched diet. Concurrent increases in *Fusobacterium* spp. and *P. hiranonis*, taxa associated with proteolytic activity and bile acid metabolism, suggest a microbial shift favoring protein and lipid fermentation pathways that may contribute to epithelial stress and inflammatory signaling. Notably, *Collinsella* spp. were also enriched during high-caloric feeding, which may be of pathophysiological relevance given previous associations of *C. aerofaciens* with hepatic inflammation, triglyceride accumulation, and pro-inflammatory signaling in high-fat diet contexts (33, 34). While the present study was not designed to establish a formal obesity model, these diet-associated microbiota alterations may help explain the inflammatory phenotype and reduced dietary tolerance observed primarily in placebo-treated animals during prolonged high-caloric feeding.

The high-caloric diet induced similar compositional and metabolic shifts in the gastrointestinal compartment across both groups, and pasteurized *A. muciniphila* Muc^T^ supplementation exerted selective effects within these baseline alterations only. While this supplementation significantly attenuated the high-caloric diet-induced expansion of *P. hiranonis* and *Collinsella* spp., it did not affect overall microbial alpha diversity or the broader community structure. This suggests that while the global microbial architecture remained largely unchanged, specific taxa with known roles in bile acid metabolism and lipid dysregulation were selectively suppressed. Similarly, no significant changes were observed in fecal lipidomic or bile acid profiles in response to the pasteurized *A. muciniphila* Muc^T^ supplementation, suggesting that this strain may not directly modulate intestinal lipid absorption as reported before in mice (35), or bile acid–microbiome metabolism under high-caloric diet conditions in canines. In murine models, however, intervention periods were consistently longer than in the present study, which was deliberately terminated before the high-caloric diet could compromise animal welfare, thereby preventing extended follow-up in an ethical manner. Nevertheless, these targeted microbial shifts, though modest, may contribute to downstream host benefits.

A limitation of the present study is the relatively small, breed-homogeneous cohort, which reduces statistical power for detecting subtle or highly variable outcomes such as body condition score and microbiota diversity metrics. Larger and more diverse cohorts may therefore be required to confirm these effects. However, given the ethical constraints associated with inducing rapid weight gain in healthy companion animals, this study was intentionally designed as an initial proof-of-concept intervention. In addition, the use of a high-caloric diet was deliberately chosen to reflect real-world overfeeding conditions in companion animals, rather than relying on more extreme experimental high-fat diets that carry known medical risks in dogs. While this approach inevitably introduced greater variability in voluntary food intake over time, it allowed improved translational relevance. In accordance with the pre-approved study protocol, a predefined go/no-go decision point was implemented at day 56 (8 weeks) to evaluate animals’ welfare, body condition, and feeding behavior. As continued high-caloric diet feeding increasingly compromised voluntary intake and body condition in control animals, the intervention was ethically terminated at this time point. Nonetheless, despite these limitations, robust and biologically coherent effects were consistently observed across multiple metabolic and inflammatory markers, supporting the biological relevance of the pasteurized *A. muciniphila* Muc^T^ induced host-response patterns.

Although the overall luminal response was dominated by the effects of the high-caloric diet, the impact of pasteurized *A. muciniphila* Muc^T^ supplementation on host inflammation was the key parameter observed in this study and was found to be linked to several health markers. Specifically, pasteurized *A. muciniphila* Muc^T^ supplementation significantly reduced levels of fecal calprotectin and was the most important feature driving separation in the multivariate analysis of host health markers. This dissociation between luminal biochemistry and mucosal health is consistent with barrier protection and immunomodulatory effects described in earlier work in mouse models, showing that both live and pasteurized *A. muciniphila* Muc^T^, as well as its outer membrane protein Amuc_1100, can restore epithelial integrity, reduce translocation of LPS, and correct gut-derived low-grade inflammation (14).

The anti-inflammatory effects of pasteurized *A. muciniphila* Muc^T^ were not restricted to the intestinal compartment. Redundancy analysis of systemic health markers demonstrated a treatment-associated separation driven primarily by differences in neutrophil- and monocyte-associated inflammatory responses, as well as TNF-α, CRP, and CXCL8. Longitudinal modeling further supported differential inflammatory trajectories over time between treatments, particularly for neutrophils, monocytes, TNF-α, and CXCL8. Together, these findings are consistent with previous reports indicating that pasteurized *A. muciniphila* may modulate host inflammatory tone through gut–immune and gut–liver interactions (13, 22). In line with prior experimental literature, host signaling pathways such as AMPK and TLR2, known to mediate epithelial repair and immune homeostasis in response to microbial ligands, have been implicated in the broader biological context of pasteurized *A. muciniphila* Muc^T^–host interactions (14, 36).

In our study, multi-omics factor analysis (MOFA) revealed that high-caloric feeding induced a coordinated host–microbiota–metabolite dysregulation characterized by systemic accumulation of microbially transformed serum bile acids (including oxidized derivatives), increased cholesterol and its oxidative marker 7-ketocholesterol, elevated inflammatory markers (TNF-α, CXCL8), higher neutrophil counts, and altered liver function tests, including alkaline phosphatase activity and albumin-globulin ratios. While this phenotype shares features with early human pre-MASH states, it is important to note that dogs do not develop MASH. Nonetheless, recent work has shown that steatotic hepatocytes in canine chronic liver disease are accompanied by innate immune activation and oxidative stress, reflecting a lipotoxic-inflammatory state that shares key hallmarks of early metabolic liver injury in humans (37). Supplementation with pasteurized *A. muciniphila* Muc^T^ significantly attenuated this dysregulation. Although intraluminal parameters such as fecal lipids, bile acids, and microbiota composition remained largely unchanged, the systemic inflammatory profile and bile acid imbalance were markedly improved.

This systemic response is compatible with a host-mediated response pattern, in which pasteurized *A. muciniphila* MucT supplementation is associated with coordinated changes in bile acid-related markers and liver-associated inflammatory readouts, without direct evidence of specific hepatic pathways. In particular, growing evidence suggests that pasteurized *A. muciniphila* improves hepatic function by engaging key regulatory and metabolic pathways. One such mechanism involves modulation of farnesoid X receptor (FXR) signaling, a central regulator of bile acid synthesis and enterohepatic feedback. In a recent study using mice with diet-induced metabolic fatty liver disease, pasteurized *A. muciniphila* Muc^T^ restored FXR–FGF15 signaling, which was associated with reduced hepatic steatosis, normalized serum bile acid pools, and improved liver integrity (38). Additionally, the strain has been shown to enhance hepatic regeneration by supporting tricarboxylic acid cycle function. A recent murine study demonstrated that treatment with live *A. muciniphila* Muc^T^ cultured on a mucus-containing medium restored tricarboxylic acid cycle intermediates (e.g., α-ketoglutarate, fumarate, and malate) and upregulated key metabolic enzymes, facilitating mitochondrial energy production and hepatocyte proliferation (39). These findings suggest a dual mechanism through which pasteurized *A. muciniphila* Muc^T^ promotes liver health, by optimizing central energy metabolism and re-establishing regulatory control over bile acid synthesis, potentially secondary to barrier restoration and reduced microbial endotoxin exposure. Recent human intervention data further support a host-directed mode of action, demonstrating improved weight-loss maintenance and favorable cardiometabolic responses following supplementation with pasteurized *A. muciniphila* Muc^T^ (40). Together with previous observations of increased fecal energy excretion in mice (35) and humans (40), these findings suggest favorable modulation of host energy balance and inflammatory signaling across species.

Altogether, these insights provide further supportive context for our observation that pasteurized *A. muciniphila* Muc^T^ mitigates diet-induced hepato-metabolic stress in dogs, not primarily through microbial remodeling, but via modulation of host pathways that integrate gut, liver, and immune function. In dogs, where obesity is common and associated with hepatic lipid accumulation and low-grade inflammation, these mechanisms may help prevent life-threatening situations. In cats, hepatic lipidosis remains a major unmet clinical need, often linked to disrupted lipid metabolism under conditions of obesity or anorexia. Although untested, the host-directed actions of pasteurized *A. muciniphila* Muc^T^ could warrant investigation in feline models, as suggested recently (41).

These observations support further exploration of pasteurized *A. muciniphila* Muc^T^ as a host-directed nutritional intervention for companion animals at risk of metabolic dysfunction and suggest that some of the immunometabolic benefits reported in rodents and humans may also extend to dogs.

## MATERIALS AND METHODS

### Preparation of pasteurized *Akkermansia muciniphila* Muc^T^

Pasteurized *Akkermansia muciniphila* Muc^T^ (ATCC BAA-835) was commercially produced as described previously (42) and standardized at a concentration of 1 B TFU per gram using microcrystalline cellulose. A daily dose of 3 grams was used.

### Rationale for high-caloric diet 2 indesign

The high-caloric diet was selected to model real-world overfeeding, which represents the primary driver of obesity in companion animals. While more tightly controlled experimental diets are described in the literature, these often rely on more extreme macronutrient shifts, such as replacement of dietary carbohydrate with very high (saturated) fat content. Such approaches are associated with substantial medical risks in dogs, including pancreatitis, and were therefore considered ethically unacceptable in healthy animals. The chosen model inevitably introduced greater variability in voluntary intake over time; however, this variability reflects real-life feeding behavior in companion animals and was explicitly monitored and accounted for in the study design and analyses.

### Animal care and treatment

This 56-day feeding trial was conducted between October 28, 2023, and December 22, 2023, in compliance with the Animal Welfare Act and following approval by the Institutional Animal Care and Use Committee (IACUC) of Summit Ridge Farms, USA. The study was designed as a proof-of-concept intervention in a limited number of clinically healthy Beagle dogs (*n* = 20; 6 males and 14 females), balancing statistical power with ethical considerations related to inducing diet-associated weight gain. Animal numbers were intentionally minimized to the smallest cohort considered sufficient to detect biologically meaningful effects while avoiding unnecessary animal use. Dogs were enrolled after a physical and blood-chemistry-based health check and manually randomized into two groups (*n* = 10 per group; each consisting of three males and seven females) based on sex, body weight, body condition score, and age, ensuring balanced distribution across treatment arms. All dogs were housed under standardized husbandry conditions at the study facility in individual enclosures compliant with the Animal Welfare Act and maintained on a 12-hour light/12-hour dark cycle. No structured exercise program, activity intervention, or differential activity regimen was implemented during the study, and all animals were managed according to the same routine husbandry procedures throughout the experimental period. Prior to study initiation, all animals were maintained on a standard colony diet, meal-fed in appropriate amounts based on the individual body condition score. The intervention was prospectively designed as a 16-week dietary challenge incorporating a predefined welfare-based go/no-go decision point at week 8 (day 56). Progression beyond this time point was contingent on acceptable body condition score values, sustained voluntary food intake, and continued weight gain without signs of physiological distress. During the intervention, continued exposure to the high-caloric diet increasingly compromised the well-being of several animals, predominantly in the control group, leading to sustained reductions in voluntary food intake and diet refusal. The study was therefore terminated at day 56 at the predefined protocol decision point to prevent progression toward excessive adiposity and undue welfare impact. As a breed-homogeneous, proof-of-concept study employing a real-world voluntary overfeeding design, the study prioritized ethical feasibility over broad population generalizability, which is explicitly acknowledged as a limitation in the Discussion.

On day 1, experimental feeding commenced, with both the control group and intervention group receiving a small portion of wet food (Cesar Filet Mignon; 40–50 g on days 1–4, and 90–100 g from day 5 onward), into which the test product was mixed for the intervention group. The wet food was offered first, and a 1-hour interval was maintained before the high-caloric dry food (Inukshuk 32/32, Corey Nutrition Company, New Brunswick, Canada) was offered. This commercial diet is designed for performance dogs and is characterized by a macronutrient composition of 32% protein, 32% fat, ~17% carbohydrate, and 3% fiber (detailed ingredient list included in the supplementary information and Table S1). The daily food quantity administered was calculated for each dog individually to provide three times the resting energy requirement (RER) using the formula 3.0 × 70 × (BW)^0.75^ (where BW is body weight in kilograms), with the goal of inducing weight gain. Dogs of the intervention group received 3 g (totaling 3 × 10e9 TFU) of pasteurized *Akkermansia muciniphila* Muc^T^ powder mixed with the wet food once daily for the duration of the 56-day feeding trial. Food was offered once daily for at least 22 h, and intake was quantified by weighing food residuals at the end of the feeding period.

### Clinical monitoring, health assessments, fecal and blood sampling

Daily clinical observations were conducted by qualified personnel to monitor adverse reactions, abnormal behaviors, coat condition, vomiting, and stool abnormalities. Veterinary physical examinations were performed at baseline and on day 56. In addition, body condition scores were recorded weekly by trained personnel using a standardized 5-point scale (1 = thin, 2 = underweight, 3 = ideal, 4 = overweight, and 5 = obese).

Food intake of the wet and dry diet was recorded daily, and body weight measurements were assessed weekly.

Stool quality was assessed twice daily on days 0, 28, and 56, and during all fresh fecal collections. Stool consistency was scored on a 0-5 scale (0 = none, 1 = watery, 2 = unformed moist, 3 = formed moist, 4 = well-formed, 5 = hard/dry).

Fresh fecal samples were collected during 3-day windows at baseline and again between days 53 and 55. Samples were immediately stored at −80°C and later shipped on dry ice to the Gastrointestinal Laboratory at Texas A&M University, USA, for DNA isolation, quantitative PCR (qPCR), 16S rRNA gene sequencing, targeted quantitative metabolomics, and calprotectin measurement.

Blood draws occurred at three time points: baseline (day 0), mid-study (day 24), and study end (day 52). Blood samples were collected via jugular venipuncture into one 2.0 mL lavender-top K2 EDTA tube and two red-top serum separator tubes (8.0 mL and 3.5 mL), following a minimum 16-hour fast. Samples were processed immediately post-collection. Serum tubes were centrifuged at 1,500 × *g* for 10 min at room temperature, and the separated serum was aliquoted into cryovials. The EDTA tubes were gently rocked for uniform mixing with the anticoagulant and not centrifuged. Samples were immediately shipped to Antech Diagnostics (USA) under low temperature-controlled conditions for blood hematology and serum chemistry; the remainder was stored at −80°C and later shipped on dry ice to the Gastrointestinal Laboratory at Texas A&M University, USA, for immunological and bile acid analyses.

### Fecal quantitative PCR of selected taxa, 16S rRNA gene sequencing and processing

DNA was extracted from 100 mg fecal material using a bead-beating method with the MoBio PowerSoil DNA isolation kit (Qiagen, Hilden, Germany).

Quantitative PCR (qPCR) was performed targeting total bacteria and a panel of 14 bacterial taxa, including *Faecalibacterium* spp., *Fusobacterium* spp., *Blautia* spp., *Turicibacter* spp., *Escherichia coli*, *Peptacetobacter (Clostridium) hiranonis*, and *Streptococcus* spp., as previously described (20). In addition, *Bacteroides* spp., *Bifidobacterium* spp., *Clostridium perfringens*, *Collinsella* spp., *Lactobacillus* spp., *Prevotella copri*, and *Ruminococcus gnavus* were quantified as previously described by Oba et al. (43), which also describes the details of all specific primers and PCR conditions used. qPCR data were expressed as log DNA abundance (fg) per 10 ng of total DNA. Reference intervals and the dysbiosis index mentioned throughout the main text were based on the study conducted by AlShawaqfeh et al. (20).

16S rRNA gene amplicon sequencing was performed as previously described by Rowe et al. (44). Briefly, DNA concentration was quantified using the Quant-iT PicoGreen dsDNA Assay Kit (Thermo Fisher Scientific, Waltham, MA, USA). The V4 region of the 16S rRNA gene was amplified using primers 515F and 806R with a dual-indexing approach. PCR reactions consisted of 2 μL 10× AccuPrime PCR Buffer II, 11.85 μL double-distilled water, 0.15 μL AccuPrime High Fidelity Taq Polymerase (Thermo Fisher Scientific), 1 μL template DNA, and 5 μL of each primer (4 μM). The amplification protocol included an initial denaturation at 95°C for 2 min, followed by 30 amplification cycles at 95°C for 20 s, annealing at 55°C for 15 s, and then 72°C for 900 s. Amplicons were sequenced on an Illumina MiSeq platform.

Raw 16S amplicon sequences were initially demultiplexed, and primer sequences were trimmed using Cutadapt v2.8 (45). Sequence quality control, denoising, and amplicon sequence variant (ASV) inference were performed using DADA2 within the QIIME2 platform (v2023.2) (46, 47). Taxonomic classification was carried out using a naïve Bayes classifier trained on the SILVA 138 reference database (48). Sequencing data are available under project PRJEB106595 in ENA.

### Fecal calprotectin

Fecal calprotectin concentrations were quantified using the Buhlmann fCAL turbo assay (BÜHLMANN Laboratories AG, Switzerland) as described previously (49). The minimum reportable value for canine samples was 3.0 μg/g. Values below this detection limit were uniformly reported as 2.9 μg/g.

### Fecal sterol and fatty acid composition

Fecal concentrations of cholesterol, cholesterol intermediates, plant sterols, and long-chain fatty acids were quantified using a targeted gas chromatography-mass spectrometry (GC-MS) approach adapted from a previously described protocol (50). Briefly, 10–14 mg of lyophilized fecal material was processed with internal standards, derivatized, and analyzed by GC-MS (Agilent 8890 GC/5977B MSD) for simultaneous detection of sterols and fatty acids. Analyte concentrations were normalized to sample dry weight and expressed as μg per mg of lyophilized feces. Full details of sample preparation, chromatographic conditions, and quantification procedures are provided by Galler et al. (50).

### Fecal and serum bile acid analyses

Fecal and serum bile acids were quantified using an in-house validated LC-MS/MS method as described by Blake et al. (51). For fecal analysis, ~100 mg of fecal material was extracted with methanol containing isotopically labeled internal standards (d_4_-glycocholic acid and d_4_-glycolithocholic acid), bead-beaten, and centrifuged at 16,000 × *g* for 10 min at 4°C. Supernatants were clarified by a second centrifugation and analyzed by LC-MS/MS. Serum bile acids were prepared following a modified extraction protocol adapted from Blake et al. (51) and validated by Martini et al. (52). Briefly, 200 µL serum was mixed with methanol containing internal standards, vortexed, centrifuged, supernatant dried under nitrogen at 65°C, reconstituted in methanol, and analyzed by LC-MS/MS.

### Blood hematology, serum chemistry, pro-inflammatory cytokines, and CRP measurement

Complete blood counts (CBC) and standard serum chemistry analyses were performed at Antech Diagnostics (USA). The CBC panel included assessment of red blood cell (RBC) count, white blood cell (WBC) count, hemoglobin (HGB), hematocrit (HCT), mean corpuscular volume (MCV), mean corpuscular hemoglobin (MCH), mean corpuscular hemoglobin concentration (MCHC), platelet count (PLT), and differential leukocyte counts. The serum chemistry panel comprised measurements of glucose, blood urea nitrogen (BUN), creatinine, total protein, albumin, globulin, alanine aminotransferase (ALT), aspartate aminotransferase (AST), alkaline phosphatase (ALP), total bilirubin, cholesterol, triglycerides, calcium, phosphorus, sodium, potassium, chloride, and bicarbonate.

All other serum markers were analyzed at the Gastrointestinal Laboratory at Texas A&M University. Concentrations of circulating pro-inflammatory cytokines were quantified using the Meso Scale Discovery (MSD) Canine Pro-Inflammatory Panel 3 (MSD, Rockville, MD, USA), a multiplex electrochemiluminescence immunoassay measuring four pro-inflammatory cytokines (IL-2, IL-6, CXCL8, and TNF-α). Assays were performed according to the manufacturer’s instructions (53), and all samples were measured in duplicate.

### Serum C-reactive protein (CRP) measurement

Serum CRP concentrations were determined using the Gentian immunoturbidimetric assay (Gentian AS, Moss, Norway) on a fully automated clinical chemistry analyzer (AU480 Chemistry Analyzer; Beckman Coulter, Brea, California, USA), following the manufacturer’s instructions. Assay principles and analytical validation are described in detail by Covin et al. (54).

### Serum C-4 and 7-KC measurements

Concentrations of 7α-hydroxy-4-cholesten-3-one (C4) and 7-ketocholesterol (7-KC) were determined as indicators of bile acid synthesis and cholesterol oxidation. The assay was analytically validated for C4 measurement with canine serum as described in supplemental materials.

### Reagents, standards, and solutions

Spectrometry-grade solvents, including acetonitrile and methanol, were purchased from VWR (Radnor, PA, USA). Analytical standards, 7α-hydroxy-4-cholesten-3-one (C4) and the isomer 3β-hydroxy-5-cholesten-7-one, also known as 7-ketocholesterol (7-KC), were obtained from Millipore Sigma (St. Louis, MO, USA). The deuterium-labeled 3β-hydroxy-5-cholesten-7-one, serving as an internal standard (IS), was obtained from C/D/N Isotopes Inc. (Pointe-Claire, Quebec, Canada). Each standard was prepared at a concentration of 1 mg/mL in 100% methanol. Subsequently, all standards underwent serial ten-fold dilution to achieve working solutions at 1,000 ng/mL. The IS spiking solution constituted a mixture of 1 µg/mL d_7_-3β-hydroxy-5-cholesten-7-one and 100% acetonitrile in a volume ratio of 5:445. All solutions were stored at −20°C.

### Instrumentation and chromatographic conditions

The LC-MS/MS assay was adapted from a published method for testing C4 in rat and monkey plasma (55). The LC-MS/MS equipment consisted of an Agilent 1260 Infinity II Binary Pump G1227B, a thermostat column compartment (G7116B), an autosampler (G7167B), and an Agilent 6470B Triple Quad mass spectrometer. Chromatographic separation was performed on a Poroshell 120 EC-C18 analytical column (2.1 × 50 mm, 2.7 µm, Agilent). Mobile phase A comprised 0.1% aqueous ammonium acetate, while mobile phase B consisted of 0.1% ammonium acetate in methanol. The gradient elution followed a sequence of 13:87 (vol/vol) mixture of A and B for 0.5 min, linear ramp from 87% to 90% B from 0.5 to 4 min, 90% to 99% B from 5 to 6 min, return to 87% B at 6.1 min, and maintenance at 87% B until the end of the run at 8.5 min. The flow rate was set at 0.3 mL/minute, with the column temperature maintained at 40°C. A 5 µL injection volume was applied.

The mass spectrometer (MS) was operated in positive electrospray ionization (ESI) mode as the ion source. Source parameters included a gas temperature at 350°C, gas flow of 10 L/min, nebulizer at 40 psi, sheath gas temperature at 400°C, and sheath gas flow of 12 L/min. The capillary and the electron multiplier voltages were set at 3,500 V and 200 V, respectively. The dynamic multiple reaction monitoring (dMRM) transitions for C4, 7-KC, and d_7_-3β-hydroxy-5-cholesten-7-one were determined using MassHunter Optimizer Software (Agilent Technologies, Germany) and are summarized in Table S2.

### Sample preparation

A mixture of 50 µL of serum and 450 µL of the IS spiking solution was vortexed for 15 s. The sample was then centrifuged at 13,000 × *g* for 20 min at 4°C. Subsequently, 200 µL of the supernatant was collected and transferred to an insert in a vial. The vial was then placed into the autosampler and maintained at 6°C.

### Statistical analyses and bioinformatics

Two animals were excluded from downstream analyses. One treatment dog consistently refused the wet diet, with or without supplementation, and thus was not exposed to the intervention. One control dog exhibited no weight gain despite adequate caloric intake, resulting in a feed conversion ratio near zero. Such resistance to diet-induced weight gain is a recognized phenomenon in rodent models, often attributed to inherent metabolic differences (16, 17). Excluding these outliers ensures the integrity of the data and the validity of the study’s conclusions.

Univariate statistical analyses were performed in R Statistical software (v4.5.0). Longitudinal weight gain and serum metabolic and inflammatory markers were assessed using linear mixed-effects models (lme4 package), with fixed effects for time, group, gender, and their interaction, and a random intercept for subject ID to account for repeated measures. Time was modeled with a 3rd-degree polynomial, as implemented in the R function poly. Due to the small number of subjects and the non-normal distribution of most variables, biomarker and clinical data were analyzed using non-parametric methods. Paired comparisons (baseline vs. endpoint) within groups were assessed using Wilcoxon signed-rank tests, and between-group comparisons were performed using Mann–Whitney *U* tests. To account for multiple hypothesis testing, *P* values were adjusted using the Benjamini–Hochberg false discovery rate (FDR) correction, where applicable. Statistical significance was defined as *P* < 0.05 (FDR-adjusted), while *P* < 0.1 was considered a statistical trend in recognition of the study’s exploratory design and small sample size. For data visualization, outcome distributions were plotted using scatterplots overlaid with Tukey-style boxplots, displaying the median, interquartile range (IQR), and whiskers extending to 1.5 × IQR. Individual data points were shown to represent variability within groups, and plots were generated using the ggplot2 package.

### Principal component analysis (PCA) and redundancy analysis (RDA)

To investigate associations between microbiota composition and metadata variables, principal component analysis (PCA) and redundancy analysis (RDA) were performed using Canoco 5.15 (56). Genus-level relative abundance tables were log-transformed according to the equation Y’, = log(1,000  ×  *Y* + 1) to normalize data distribution prior to ordination. All other parameters, such as health markers and bile acid composition, were normalized via *p*' = *P*/sum(*P*), where sum *p’* is the normalized parameter and *P* is the parameter for a specific sample.

### Hierarchical all-against-all association (HAllA)

To identify statistically significant associations between microbial features and host variables, hierarchical all-against-all association (HAllA) analysis was performed using v0.8.40 of the HAllA software, as previously described (57). Associations were computed using Spearman correlation. Multiple testing correction was performed using the Benjamini–Hochberg method with a false discovery rate threshold of 0.1. The analysis included permutation testing to assess empirical significance and generated clustered heatmaps for visualization of association patterns.

### Multi-omics factor analysis (MOFA)

Integration of multi-omics data sets was performed using the MOFA2 framework, implemented via the R package MOFA2 v1.16.0, as previously described by Argelaguet et al. (58). Prior to model training, data matrices were transformed using the Box-Cox method to stabilize variance and improve normality of the data, thereby reducing the impact of outliers and improving comparability across omics data sets. Box-Cox transformations were applied using the SciPy package (v1.15.1, https://docs.scipy.org) in Python. Default MOFA2 settings were used.

## Data Availability

Sequencing data generated in this study have been deposited in the European Nucleotide Archive under project accession number PRJEB106595.

## SUPPLEMENTAL MATERIAL

Fig. S1 spectrum.03226-25-s0001.tif Body condition score after 8 weeks of high-caloric diet intake and *A. muciniphila* MucT treatment.

Fig. S2 spectrum.03226-25-s0002.tif High-caloric diet feeding alters fecal sterol, fatty acid, and bile acid composition.

Fig. S3 spectrum.03226-25-s0003.tif Alpha diversity metrics of microbial communities stratified by time point (T0, T8) and treatment group (control, test).

Fig. S4 spectrum.03226-25-s0004.tif High-caloric diet intake alters gut bacterial taxa as measured by qPCR.

Fig. S5 spectrum.03226-25-s0005.tif Associations between *Peptacetobacter hiranonis* and the fecal bile acid profile.

Fig. S6 spectrum.03226-25-s0006.tif Redundancy analysis (RDA) of diet-induced microbiota shifts and their association with food intake and inflammatory markers.

Fig. S7 spectrum.03226-25-s0007.tif Heatmap of the principal serum bile acids, metabolic markers, and immune-related parameters contributing to MOFA Factor 1.

Fig. S8 spectrum.03226-25-s0008.tif Bile acid metabolic pathways in gut and liver and log2 fold changes from baseline to week 8 in serum levels of bile acids between control and *Akkermansia muciniphila* MucT–treated animals.

Supplemental material spectrum.03226-25-s0009.docx Supplemental results and legends for Fig. S1 to S8.

ASM does not own the copyrights to Supplemental Material that may be linked to, or accessed through, an article. The authors have granted ASM a non-exclusive, world-wide license to publish the Supplemental Material files. Please contact the corresponding author directly for reuse.
