# Supplementary material for: Pasteurized Akkermansia muciniphila MucT reduces high-caloric diet-induced weight gain and alters bile acid and inflammatory profiles in dogs
Source: Microbiol Spectr. 2026 Jul 14;14(8):e03226-25. doi: 10.1128/spectrum.03226-25 (PMC13436404; doi:10.1128/spectrum.03226-25)
Supplement: Supplementary file 1 [file spectrum.03226-25-s0009.docx]

**SUPPLEMENTAL MATERIAL**

**Supplementary Table 1.** Inukshuk 32/32 sport’s diet macronutrient composition (Metabolizable Energy (ME) = 4505 kcal/kg)

| **Nutrient** | **Guaranteed** | **Percentage** |
| --- | --- | --- |
| **Crude Protein** | **Min.** | **32.00%** |
| **Crude Fat** | **Min.** | **32.00%** |
| **Crude Fibre** | **Max.** | **3.00%** |
| **Moisture** | **Max.** | **10.00%** |

**Ingredients**:

Chicken Meal, Chicken Fat, Herring Meal, Ground Whole Grain Corn, Ground Whole Grain Wheat, Wheat Shorts, Dried Beet Pulp, Dried Brewer’s Yeast, Herring Oil, Salt, Chicken Liver, Kelp Meal, Potassium Chloride, Lecithin, Chicory Root, Mixed Tocopherols (preservative), Flaxseed, Glucosamine Hydrochloride, Chondroitin Sulfate, Black Malted Barley, Calcium Propionate (preservative), Limestone, Iron Proteinate, Zinc Proteinate, L-Ascorbyl-2-Polyphosphate (Vitamin C), Vitamin E Supplement, Taurine, Manganese Proteinate, Yucca Schidigera Extract, Copper Proteinate, L-Lysine, Selenium Yeast, Vitamin A Supplement, Niacin (B3), Calcium Pantothenate (B5), Biotin (B7), Riboflavin (B2), Thiamine Mononitrate (B1), Vitamin B12 Supplement, Pyridoxine Hydrochloride (B6), Vitamin D3 Supplement, Cobalt Sulfate, Calcium Iodate, Folic Acid (B9)

**Supplementary Table 2.** Monitored ion transitions and chromatographic retention times of 7α-hydroxy-4-cholesten-3-one (C4), 3β-hydroxy-5-cholesten-7-one (7-ketocholesterol; 7-KC), and d7-3β-hydroxy-5-cholesten-7-one and their parameter settings.

| Compound | Retention time (min) | Precursor ion | Product ion | Fragmentor Voltage | Collision Energy |
| --- | --- | --- | --- | --- | --- |
| C4 | 3.2 | 401.3 | 383.3 | 154 | 16 |
|  |  |  | 365.3 | 154 | 20 |
|  |  |  | 177.1 | 154 | 24 |
| 7-KC | 3.9 | 401.3 | 383.3 | 154 | 16 |
|  |  |  | 177.1 | 154 | 24 |
| d_7_-3β-hydroxy-5-cholesten-7-one | 3.8 | 408.4 | 390.3 | 144 | 27 |
|  |  |  | 95.1 | 155 | 35 |

**C4 Method validation**

For specificity testing, an isomer of 7α-hydroxy-4-cholesten-3-one (C4), namely 3β-hydroxy-5-cholesten-7-one, also known as 7-ketocholesterol (7-KC), was incorporated into the assay. To evaluate linearity, a calibration range was established from 1.95 to 1000 ng/mL with ten concentration levels run in duplicate. Working solutions of C4 and 7-KC at a concentration of 2 μg/mL were combined in equal volumes to form the highest concentration of the standard curve. All calibrators were prepared using methanol through a 2-fold serial dilution of the working solutions. Carryover was evaluated by examining both a no-injection blank and a blank sample consisting of 100% methanol immediately following the completion of the run involving the highest concentration standard. Employing the calibration curve and a signal-to-noise (S/N) ratio, the lower limit of quantification (LLOQ) was determined. To assess the precision and accuracy of the assay, inter- and intra-assay variations were examined by testing five canine serum samples covering low to high concentrations across five different days and injecting samples six times within the same day. To evaluate the matrix effect, five canine serum samples covering low, medium and high concentrations were subjected to 1:1, 1:4, and 1:9 dilutions (serum/water: v/v) pre-extraction with water. Additionally, canine serum samples covering low, medium and high concentrations were spiked with a standard of 250 ng/mL C4 and 7-KC.

**Results**

The assay was specific for C4 and 7-KC because neither compound contributed to the other’s signal, and they were baseline resolved chromatographically. For linearity assessment, the calibration curve, incorporating ten concentrations of standards (1.95-1000 ng/mL), was evaluated and all ten concentration levels fell within 20% of expected value and had S/N ratio above 10. Carryover testing resulted in <20% of the lowest concentration standard being present in the blanks following the highest concentration standard injection. The LLOQ was 1.95 ng/mL and the mean (SD) concentration resulting from ten quantifications at this level was 2.09 ± 0.21 ng/mL with S/N ratios of 32 ± 9.

For canine serum, the medians (range) of observed-to-expected (O/E) ratios at 2-, 5-, and 10-times dilutions were 100.9% (83.3-108.6), 98.7% (92.1-113.8), and 93.6% (90.1-153.1), respectively. Notably, a sample with an original concentration of 19.6 ng/mL, when diluted 10 times, exhibited a high O/E ratio, resulting in 3 ng/mL. Theoretically, the sample with a low concentration did not necessitate dilution. The medians (range) of the coefficients of variation for intra-assay and inter-assay were 4.2% (1.4-7.9) and 12.1% (7.4-14.8), respectively. The median (range) of O/E ratio of canine samples spiked with a standard was 113.3% (104.4-126.1).

**Supplementary Figure 1. Body condition score after 8 weeks of high-caloric diet intake and *A. muciniphila* Muc^T^ treatment.** Body condition scores were analysed after 8 weeks of treatment 1: Thin 2: Underweight 3: Ideal 4: Overweight 5: Obese. Violin plots illustrate the distribution of body condition scores (BCS) in the *A. muciniphila* Muc^T^ group (blue) and controls (red), with overlaid boxplots (Tukey-style) and individual data points (n = 9 per group). A one-sided Wilcoxon rank-sum test was performed to evaluate the hypothesis that the BCS in the *A. muciniphila* Muc^T^ group was lower than in the control group, resulting in p = 0.09 (trend).

**Supplementary Figure 2. High-caloric diet feeding alters fecal sterol, fatty acid and bile acid composition**. (A) Eight weeks of high-caloric diet feeding induced marked shifts in the fecal fatty acid profile. Redundancy analysis revealed significant reductions in plant-derived sterols (campesterol, fucosterol, stigmasterol, β-sitosterol) and polyunsaturated fatty acids (linoleic acid, α-linolenic acid), while cholesterol, its microbial metabolites (cholestanol, coprostanol), saturated long-chain fatty acids (myristic acid [C14:0], stearic acid [C18:0]) and nervonic acid were increased. (B) The high-caloric diet also induced altered fecal bile acid composition. Redundancy analyses indicated increased fecal levels of most bile acids induced by the high-caloric diet, when compared to baseline samples, potentially reflecting increased bile acid synthesis and microbial bile acid metabolism. Large triangles are centroids of the sample groups (Time * Treatment), while the other symbols indicate individual samples. Ellipses are the 66% quantile of the approximated 2D-normal density distribution function for each sample group.

**Supplementary Figure 3. Alpha diversity metrics of microbial communities stratified by Timepoint (T0, T8) and Treatment group (Control, Test).** Each point represents an individual sample, colored by treatment and grouped by timepoint. Diversity metrics shown include Pielou's evenness, Faith's Phylogenetic Diversity (PD), Observed Features (richness), and Shannon entropy. These metrics capture different aspects of microbial diversity, including richness, evenness, and phylogenetic breadth. Boxplots indicate the means and standard-deviation range per group. Differences in diversity across timepoints and treatments reflect potential microbiome shifts associated with intervention and time (high-caloric).

**Supplementary Figure 4. High-caloric diet intake alters gut bacterial taxa as measured by qPCR**. Quantitative PCR (qPCR) analysis revealed significant shifts in log DNA levels of selected bacterial taxa in response to high-caloric diet feeding. For clarity, only data from the control group are shown. Paired t-tests were used to compare baseline (week 0) and post-diet (week 8) abundances, with P-values adjusted for multiple testing. Results are expressed as log10 DNA copy numbers per gram feces. Statistical significance is indicated as: p < 0.05 (*), p < 0.01 (**), p < 0.001 (***), p < 0.0001 (****).

**Supplementary Figure 5. Associations between *Peptacetobacter hiranonis* and the fecal bile acid profile.** (A) Redundancy analysis (RDA) revealed that *P. hiranonis*, a key 7α-dehydroxylating bacterium in dogs, was strongly associated with fecal bile acid composition (p = 0.002; 16.8% explained variation). Its abundance correlated positively with secondary bile acids [lithocholic acid (LCA), deoxycholic acid (DCA)], their oxidized derivatives (12-oxo-LCA, 3-oxo-DCA), and taurine-conjugated bile acids (TUDCA, TDCA, TCDCA, TLCA), supporting a role in shaping the secondary bile acid pool under dietary lipid stress. RDA was performed with fecal bile acids as response variables and *P. hiranonis* qPCR values as explanatory variable (red arrow). (B) HAllA analysis further demonstrated hierarchical associations between *P. hiranonis* and clusters of bile acids. Rows represent bacterial taxa and columns bile acids, with hierarchical clustering applied to both datasets. Color intensity reflects Spearman correlation (red = positive, blue = negative), and statistically significant blocks of associations (FDR alpha = 0.1) are delineated by rectangles. Numbers within blocks indicate the rank order of significance, with “1” representing the most significant association (lowest adjusted p-value).

**Supplementary Figure 6**. **Redundancy analysis (RDA) of diet-induced microbiota shifts and their association with food intake and inflammatory markers.** RDA ordination plot (P = 0.028) showing the relationship between the fold-change in bacterial genera from baseline (T0) to week 8 (T8) under a high-calorie (HC) diet and food intake during the same week of fecal sampling (week 8). Genera with the strongest contribution to the ordination are displayed as vectors. Supplementary variables include pro-inflammatory immune markers and *Fusobacterium* abundance as determined by qPCR. Notably, the *Fusobacterium* qPCR vector aligns with the direction of 16S-based *Fusobacterium* abundance, indicating concordant explanatory patterns between sequencing and targeted quantification approaches. The analysis reveals that lower food intake is associated with a decline in *Lactobacillus* and *Ruminococcus* spp., and an increase in proteolytic taxa like *Fusobacterium* and *Clostridium* sensu stricto 13, both of which align with inflammatory markers, suggestive of a potential feedback loop between inflammation, microbial dysbiosis, and appetite suppression.

**Supplementary Figure 7. Heatmap of the principal serum bile acids, metabolic markers, and immune-related parameters contributing to MOFA Factor 1.** Heatmap showing the longitudinal profiles of the variables with the largest weights in Factor 1 of the Multi-Omics Factor Analysis (MOFA), including serum bile acids (top panel) and systemic metabolic and immune-related parameters (bottom panel). Columns represent individual dogs grouped by treatment (Control or *Akkermansia muciniphila* Muc^T^) and sampling time point Week 0 (W0) and 8 (W8). Heatmap colours represent input for the MOFA analysis, with darker shades indicating relatively higher values and lighter shades indicating relatively lower transformed values within each feature. The accompanying bar plots indicate the corresponding Factor 1 feature weights, where positive weights denote variables contributing positively to Factor 1 and negative weights denote variables contributing inversely.

**Supplementary Figure 8. Bile acid metabolic pathways in gut and liver and log2fold changes from baseline to week 8 in serum levels of bile acids between control and *Akkermansia muciniphila* Muc^T^ treated animals**. Secondary bile acids, deoxycholic acid (DCA) and lithocholic acid (LCA), are absorbed into enterohepatic circulation and re-conjugated with taurine and glycine in the liver. Violin plots pasted into the diagram are the Log2Fold changes from 0 to week 8, by treatment. Red violins are control group and teal violins are Akkermansia treated group with green horizontal bars representing zero log2 fold change. P-values are FDR corrected. Diagram used and modified with permission from: Altered iso- and oxo-Fecal Bile Acid Concentrations in Dogs with Chronic Enteropathy. Serum and fecal markers in red text indicated next to bile acids represent significant positive correlations in abundance (identified by HAIIA), illustrating shifts in bile acid metabolism and associated host markers. Abbreviations: BSH, bile salt hydrolase; HSDH, hydroxysteroid dehydrogenase; bai, (bile acid-inducible) bai genes for 7α-dehydroxylation enzymes.
